# Hypothesis Testing With Rank Conditions in Phylogenetics

**DOI:** 10.3389/fgene.2021.664357

**Published:** 2021-07-02

**Authors:** Colby Long, Laura Kubatko

**Affiliations:** ^1^Department of Mathematical and Computational Sciences, College of Wooster, Wooster, OH, United States; ^2^Department of Statistics and Evolution, Ecology, and Organismal Biology, The Ohio State University, Columbus, OH, United States

**Keywords:** SVDquartets, ErikSVD, flattening matrix, multispecies coalescent, singular value decomposion

## Abstract

A phylogenetic model of sequence evolution for a set of *n* taxa is a collection of probability distributions on the 4^*n*^ possible site patterns that may be observed in their aligned DNA sequences. For a four-taxon model, one can arrange the entries of these probability distributions into three flattening matrices that correspond to the three different unrooted leaf-labeled four-leaf trees, or quartet trees. The flattening matrix corresponding to the tree parameter of the model is known to satisfy certain rank conditions. Methods such as ErikSVD and SVDQuartets take advantage of this observation by applying singular value decomposition to flattening matrices consisting of empirical data. Each possible quartet is assigned an “SVD score” based on how close the flattening is to the set of matrices of the predicted rank. When choosing among possible quartets, the one with the lowest score is inferred to be the phylogeny of the four taxa under consideration. Since an *n*-leaf phylogenetic tree is determined by its quartets, this approach can be generalized to infer larger phylogenies. In this article, we explore using the SVD score as a test statistic to test whether phylogenetic data were generated by a particular quartet tree. To do so, we use several results to approximate the distribution of the SVD score and to give upper bounds on the *p*-value of the associated hypothesis tests. We also apply these hypothesis tests to simulated phylogenetic data and discuss the implications for interpreting SVD scores in rank-based inference methods.

## 1. Background on Phylogenetics and SVDQuartets

Recent technological advances have reduced both the time and the cost required to obtain DNA sequence data from biological samples. The widespread availability of large-scale data sets has necessitated the development of methods that can efficiently estimate the evolutionary relationships among the samples as represented by a phylogenetic tree. Because traditional frameworks for statistical inference, such as the maximum likelihood and Bayesian frameworks, require thorough searches of tree space in order to provide estimates, they become computationally prohibitive when the size of the data is large and/or when estimation under a complex model, such as the multispecies coalescent, is desired. Several approaches for estimation that don't require computation of the likelihood while still being model-based have recently been proposed (Eriksson, 2005; Chifman and Kubatko, 2014) and represent promising alternative methods for inferring phylogenies when the data size is large.

**Figure 1:**
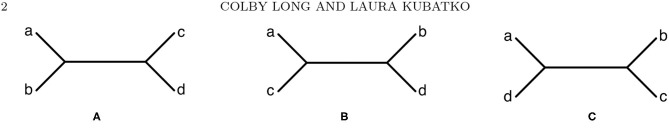
The three possible unrooted tree topologies for four taxa. **(A)** Tree *T*_1_, **(B)** Tree *T*_2_, and **(C)** Tree *T*_3_.

To understand how these approaches work, we begin by defining the probability distribution of the data arising along a phylogeny. As an example, consider the four-taxon tree shown in Figure 1A, and define a site pattern *i*_1_*i*_2_*i*_3_*i*_4_ as an assignment of states to the tips of the tree, i.e., *i*_1_ is the state assigned to taxon *a*, *i*_2_ is the state assigned to taxon *b*, etc. Here we consider DNA sequence data, so that *i*_*j*_ ∈ {*A, C, G, T*} for *j* = 1, 2, 3, 4. For a tree that includes branch lengths together with a model by which the mutation process occurs along the tree, let *p*_*i*_1_*i*_2_*i*_3_*i*_4__ refer to the probability of observing site pattern *i*_1_*i*_2_*i*_3_*i*_4_ under the chosen model. Note that a total of 4^4^ = 256 site patterns are possible for a four-taxon tree. The collection *p* of these 256 site pattern probabilities will be referred to as a *site pattern probability distribution*.

This probability distribution can be arranged into a 16 × 16 matrix, referred to as a *flattening*, such that the rows of the matrix correspond to possible states for one pair of sister taxa in the tree and the columns of the matrix correspond to possible states for the two remaining taxa. For example, for tree *T*_1_ in Figure 1A, the flattening matrix *F*_*T*_1__ (*p*) is given by

$$\begin{array}{l} F_{T_{1}}\left(p\right)=\left(\begin{array}{cccccccc} \; & \left[AA\right] & \left[AC\right] & \left[AG\right] & \left[AT\right] & \left[CA\right] & \cdots & \left[TT\right]\\ \left[AA\right] & p_{AAAA} & p_{AAAC} & p_{AAAG} & p_{AAAT} & p_{AACA} & \cdots & p_{AATT}\\ \left[AC\right] & p_{ACAA} & p_{ACAC} & p_{ACAG} & p_{ACAT} & p_{ACCA} & \cdots & p_{ACTT}\\ \left[AG\right] & p_{AGAA} & p_{AGAC} & p_{AGAG} & p_{AGAT} & p_{AGCA} & \cdots & p_{AGTT}\\ \left[AT\right] & p_{ATAA} & p_{ATAC} & p_{ATAG} & p_{ATAT} & p_{ATCA} & \cdots & p_{ATTT}\\ \left[CA\right] & p_{CAAA} & p_{CAAC} & p_{CAAG} & p_{CAAT} & p_{CACA} & \cdots & p_{CATT}\\ \cdots & \cdots & \cdots & \cdots & \cdots & \cdots & \cdots & \cdots \\ \left[TT\right] & p_{TTAA} & p_{TTAC} & p_{TTAG} & p_{TTAT} & p_{TTCA} & \cdots & p_{TTTT} \end{array}\right) \end{array}$$

where, for example, the (5, 3) entry, *p*_*CAAG*_, refers to the probability that taxon *a* has nucleotide *C*, taxa *b* and *c* have nucleotide *A*, and taxon *d* has nucleotide *G*. Note that it is also possible to construct flattening matrices for the other two trees in Figure 1, *F*_*T*_2__ (*p*) and *F*_*T*_3__ (*p*), where *p* is the probability distribution derived under the assumption that *T*_1_ is the true phylogeny.

Previous work has examined the properties of the flattening matrices *F*_*T*_*b*__ (*p*), *b* = 1, 2, 3, under various evolutionary models. In the case in which DNA sequence data are assumed to have evolved along a single phylogenetic tree (the gene tree *T*_1_), Allman and Rhodes (2006) showed that *F*_*T*_1__ (*p*) is generically rank 4, while the matrices *F*_*T*_2__ (*p*) and *F*_*T*_3__ (*p*) are generically rank 16, under a variety of models for the DNA substitution process that includes the general time reversible (GTR) model (Liò and Goldman, 1998). Chifman and the second author (2015) considered the case in which sequence data arise under the multispecies coalescent model (Edwards et al., 2016; Kubatko, 2019) from a species tree with topology matching *T*_1_ but with the root placed along the internal branch of the tree so that the tree satisfies the molecular clock. They showed that in this case *F*_*T*_1__ (*p*) is generically rank 10, while the other two flattening matrices generically have rank strictly greater than 10, for the GTR+I+Γ model and all submodels. The authors generalized this result to the case in which the population size and/or mutation rate varies for any submodel of the GTR model, thus establishing a reduced-rank result for species trees under the multispecies coalescent even in the absence of a molecular clock (Long and Kubatko, 2019). More recent results have shown that if *p* is a generic probability distribution from a network model of evolution where the network has a tree clade with species *a* and *b* or *c* and *d*, then *F*_*T*_1__ (*p*) will be rank 4 (Casanellas and Fernández-Sánchez, 2020).

These results suggest a method for inferring phylogenetic trees under a wide range of models. Specifically, the probability distribution *p* can be approximated for a given data set using the observed frequencies of the site patterns, the collection of which we denote by $\hat{q}$. For an alignment of length *n*, the estimated probability distribution $q=\hat{q}/n$ can then be used to form three estimated flattening matrices corresponding to the three trees in Figure 1. A measure of how close each of the estimated flattening matrices is to the nearest matrix of the relevant rank (e.g., rank 4 when the goal is to infer the gene tree, and rank 10 when the goal is to infer the species tree) can then be used to infer the four-taxon tree by picking that for which the corresponding matrix is closest to the desired rank. For data sets containing more than four taxa, clustering methods or quartet assembly procedures can be used to obtain an overall estimate of the phylogeny from a set of inferred quartet relationships. These approaches are implemented in the ErikSVD software (Eriksson, 2005) in the case of gene trees and in the SVDQuartets software [part of the PAUP* package (Swofford, 2021)] in the case of species trees. As their names imply, both methods use singular value decomposition (SVD) to compute an *SVD score*, the distance between the flattening matrices and the appropriate set of reduced-rank matrices. We provide the rationale for use of the SVD score as well as the details of its computation in the next section.

While we have described the concept of a flattening matrix using four-taxon trees, such matrices can be constructed for larger trees as well. To do this, we consider cutting an internal branch of a tree, which splits the taxa at the tips of the tree into two sets, *L*_1_ and *L*_2_. The flattening matrix corresponding to this split of taxa is then constructed by letting the rows of the $4^{\left|L_{1}\right|}\times 4^{\left|L_{2}\right|}$ matrix correspond to possible nucleotides for the taxa in *L*_1_ and the columns to possible nucleotides for the taxa in *L*_2_. While reduced rank results analogous to those described above are known for gene trees (see e.g., Allman and Rhodes, 2007), no such results are available for species trees for more than four taxa. Our focus in the remainder of this paper will be on four-taxon trees, as these form the building blocks for inference under a large class of models.

SVD-based methods have proven remarkably effective (see e.g., Chifman and Kubatko, 2014; Wascher and Kubatko, 2021) at accurately inferring the correct quartet tree from model data. Thus, far, however, they have only been used as a means of estimating the true quartet topology, rather than as a measure of confidence that a particular quartet topology is the one that gave rise to the observed data. Here we explore the question of whether the magnitude of the SVD score can be used to assess support in the data for the quartet tree underlying the flattening matrix from which it has been computed. Such an assessment has many applications. For example, it could be used to improve the performance of inference methods like ErikSVD and SVDQuartets by providing weights for the quartet trees in proportion to their support in the data in order to allow downstream analyses to capture more of the information contained in the quartet data. As a first step in this direction, in this work we use the SVD score to construct a hypothesis test of the null hypothesis that the data arose from a particular quartet tree. If we view a single quartet tree in isolation, the test we develop is a formal hypothesis test of whether we may reject this tree. However, we note that this test cannot be applied simultaneously to the three SVD scores from a rank-based quartet inference method. Indeed, this would require developing a test based on the joint distribution of the SVD scores of the three flattening matrices. Still, a better understanding of the distribution of the SVD score will reveal more about why these methods are so effective and enable some principled decisions for weighting quartets.

We begin in section 2 by defining the SVD score and describing what precisely it measures. In section 3.1, we present results describing the distribution of the SVD score and in section 3.2 we derive probabilistic bounds on its magnitude. Our results allow us reject the null hypothesis that the data arose from a specified quartet tree when the SVD score exceeds a cut-off based on these bounds. In section 4, we apply our hypothesis test to data simulated from some commonly-used phylogenetic models, and obtain estimates of the number of sites required to reject a *discordant* quartet, one that does not agree with the quartet that generated the data. We also reveal some surprising results about the inner workings of SVD-based methods that suggest the results should be interpreted carefully. Finally, in section 5 we discuss the implications and applications of these results for SVD-based inference methods.

## 2. The SVD Score

As described above, the main tool needed to enable inference of phylogenies from the estimated site pattern probability distribution *q* is a measure of the distance of the flattening matrix corresponding to each of the trees in Figure 1 to the nearest matrix of the relevant rank, *r*. To define this measure, consider a *u* × *v* matrix *A* with (*i, j*)^*th*^ entry *a*_*ij*_. The *Frobenius norm* of matrix *A* is given by

$$\begin{array}{l} \|A{\|}_{F}=\sqrt{\overset{u}{\underset{i=1}{\sum }}\overset{v}{\underset{j=1}{\sum }}a_{ij}^{2}}=\sqrt{\overset{s}{\underset{i=1}{\sum }}\sigma_{i}^{2}}, \end{array}$$

where σ_1_ ≥ σ_2_ ≥ ⋯ ≥ σ_*s*_ ≥ 0 are the *singular values* of *A* and *s* = min{*u, v*}.

The Eckart-Young Theorem (Eckart and Young, 1936) tells us that the distance from the matrix *A* to the nearest rank *r* matrix under the Frobenius norm is

$$\begin{array}{l} \underset{\text{rank}\left(B\right)=r}{\min}\|A-B{\|}_{F}=\sqrt{\overset{s}{\underset{i=r+1}{\sum }}\sigma_{i}^{2}}. \end{array}$$

We use this result as the basis for the SVD score for tree *T* ∈ {*T*_1_, *T*_2_, *T*_3_} based on the estimated flattening matrix *F*_*T*_(*q*). Specifically, the SVD score for tree *T* and rank *r* is defined to be

(1) $$\begin{array}{l} S_{r}^{T}\left(q\right)=\sqrt{\overset{16}{\underset{i=r+1}{\sum }}{\hat{\sigma }}_{i}^{2}} \end{array}$$

where $\hat{\sigma_{i}}$ is the *i*^*th*^ singular value of *F*_*T*_(*q*).

The intuition behind the SVD score is that a *u* × *v* matrix of rank *r* < *s* will have σ_*i*_ = 0 for *i* = *r* + 1, *r* + 2, ⋯ , *s*. Because the flattening matrices *F*_*T*_(*q*) are computed based on the estimated site pattern probability distribution *q* rather than on the true probability distribution *p*, they will all be full rank, even for the true tree *T*_1_. However, the (*r* + 1)^*st*^ through *s*^*th*^ singular values are expected to be closer to 0 for the true tree than for the two alternative trees, and thus small values of the SVD score indicate better fit of the data to the tree. The algorithms underlying both ErikSVD and SVDQuartets are based on the selection of the smallest SVD score from among a set of alternatives in order to successively build the phylogenetic estimate of the true tree.

One important point to note is that the SVD score gives the distance to the nearest rank *r* matrix from among the set of all rank *r* matrices, which we denote by $F_{r}^{T}$. While this is a useful measure of the support in the data for a particular phylogeny, as we demonstrate below, it is not the most appropriate measure that could be conceived. For example, a more appropriate measure would be the distance to the nearest rank *r* matrix that encodes a probability distribution; an even more appropriate measure would be the distance to the nearest rank *r* matrix that encodes a probability distribution that arises from a specific model for DNA sequence evolution on a phylogenetic tree. We call such a model a *phylogenetic model* and use M to denote the set of probability distributions in the model. However, the computation of distances in these scenarios is non-trivial, and the development of efficient methods for computing such distances is an open challenge. Thus, we consider here the properties of the SVD score in Equation (1) instead, as it can be rapidly computed even when the sequence length *n* is large. In addition, the SVD score gives a lower bound on the other distances that we mention above, a fact which we use in our hypothesis tests.

## 3. Hypothesis Testing With the SVD Score

The theory above was described using the estimated site pattern probability distribution *q*. However, in order to develop the statistical theory underlying our proposed hypothesis tests, it will be convenient for us to use instead the observed site pattern frequency vector $\hat{q}$ in the SVD score. All of the theory above remains essentially unchanged. If we have an observed alignment of length *n*, then the flattening matrix $F_{T}\left(\hat{q}\right)=nF_{T}\left(q\right)$ and the SVD score $S_{r}^{T}\left(\hat{q}\right)=nS_{r}^{T}\left(q\right)$ is the Euclidean distance from the observed site pattern frequency vector $\hat{q}$ to the set $F_{r}^{T}$ of rank *r* flattening matrices.

Our goal will be to use the SVD score to test the null hypothesis that $\hat{q}$ is a sample from a multinomial distribution that is contained inside a model $M\subseteq F_{r}^{T}$. More specifically, we will develop a hypothesis test in which the squared SVD score serves as an upper bound on the test statistic. In this section, we introduce the test statistic and outline our approach.

Let *X*^(*n,p*)^ be the vector-valued random variable that records the number of occurrences of *i* after *n* draws from the multinomial distribution *p* ∈ Δ^*k*−1^ and define

$$Y^{\left(n,p\right)}=\overset{k}{\underset{i=1}{\sum }}{\left(X_{i}^{\left(n,p\right)}-p_{i}n\right)}^{2}.$$

Thus, *Y*^(*n,p*)^ is the squared Euclidean distance between the expected and observed frequency vector. In the context of phylogenetics, the observed frequency vector is a vector of site pattern counts obtained from a DNA sequence alignment of *n* sites. In the case we consider, where the phylogenetic model is a 4-state DNA substitution model for four species, *k* = 256. Given a site pattern probability distribution *p*, we may now use *Y*^(*n,p*)^ as a test statistic to test the null hypothesis

$H_{0}:\hat{q}$ is a sample distribution obtained by drawing *n* sites from *p*.

The *p*-value associated with this test is then $Pr\left(Y^{\left(n,p\right)}\geq \|pn-\hat{q}{\|}_{2}^{2}\right)$, where || · ||_2_ is the Euclidean or ℓ_2_ norm.

For our purposes, we are most interested in the case where we do not know *p* explicitly, but instead only know that *p* belongs to a phylogenetic model $M\subseteq F_{r}^{T}$. In this case, while we do not know the exact distance between *pn* and $\hat{q}$, the SVD score gives us a lower bound on this distance. That is, $S_{r}^{T}\left(\hat{q}\right)\leq \|pn-\hat{q}{\|}_{2}$. Therefore, in terms of our hypothesis test, we can obtain an upper bound on the *p*-value since

$$Pr\left(Y^{\left(n,p\right)}\geq \|pn-\hat{q}{\|}_{2}^{2}\right)\leq Pr\left(Y^{\left(n,p\right)}\geq {\left(S_{r}^{T}\left(\hat{q}\right)\right)}^{2}\right).$$

Thus, if we choose significance level α for our hypothesis test, then we can safely reject the null hypothesis when $Pr\left(Y^{\left(n,p\right)}\geq {\left(S_{r}^{T}\left(\hat{q}\right)\right)}^{2}\right)<\alpha$.

For phylogenetic inference, rather than testing whether our data were generated by a particular distribution, we are interested in whether our data were generated by a particular phylogenetic model, M. Thus, we wish to test the null hypothesis,

$H_{0}:\hat{q}$ is a sample distribution obtained by drawing *n* sites from some $p\in M\subseteq F_{r}^{T}$.

Then we may reject the null hypothesis when we are able to reject that the data are a sample from any distribution in the model, that is to say, when

$$\left(\underset{p\in M}{\max}Pr\left(Y^{\left(n,p\right)}\geq \|pn-\hat{q}{\|}_{2}^{2}\right)\right)<\alpha .$$

By the same reasoning as above, this allows us to reject the null hypothesis whenever

$$\left(\underset{p\in M}{\max}Pr\left(Y^{\left(n,p\right)}\geq {\left(S_{r}^{T}\left(\hat{q}\right)\right)}^{2}\right)\right)<\alpha ,$$

And of course, since $M\subseteq \Delta^{k-1}$, this means we can reject whenever

$$\left(\underset{p\in \Delta^{k-1}}{\max}Pr\left(Y^{\left(n,p\right)}\geq {\left(S_{r}^{T}\left(\hat{q}\right)\right)}^{2}\right)\right)<\alpha ,$$

where now the maximum is taken over the entire simplex. In order to leverage this observation, we now require results on the distribution of *Y*^(*n,p*)^.

### 3.1. The Distribution of *Y*^(*n,p*)^

For sufficiently large *n* the distribution of *X*^(*n,p*)^ is approximated by the multivariate normal distribution N(np,nΣ(p)), where

$$\Sigma {\left(p\right)}_{ij}=\{\begin{array}{ll} p_{i}\left(1-p_{i}\right) & i=j\\ -p_{i}p_{j} & i\neq j. \end{array}$$

Thus, the random variable *X*^(*n,p*)^ − *np* is approximated by N(0,nΣ(p)) (Wasserman, 2010). Since Σ(*p*) is a real symmetric matrix, we can use the spectral decomposition to write Σ(*p*) = *Q*^*T*^Λ*Q*, where Λ is a diagonal matrix of the eigenvalues of Σ(*p*) and *Q*^*T*^*Q* = *I*. Moreover, since Σ(*p*) is positive semidefinite, all of its eigenvalues are non-negative real numbers and so we may take the square root of Λ and write Σ(*p*)^1/2^ = *Q*^*T*^Λ^1/2^*Q*. Although our primary interest is when *p* is a probability distribution, we will assume for the moment that 0 < *p*_*i*_ < 1 and $\sum_{i=1}^{k}p_{i}<1$. In this case, Σ(*p*) is invertible (Withers and Nadarajah, 2014) and so Σ(*p*)^−1/2^ = *Q*^*T*^Λ^−1/2^*Q*. Then we may write

$$\begin{array}{l} Y^{\left(n,p\right)}={\left(X^{\left(n,p\right)}-np\right)}^{T}\left(X^{\left(n,p\right)}-np\right)\\ \;={\left(X^{\left(n,p\right)}-np\right)}^{T}\Sigma {\left(p\right)}^{-1/2}\left(Q^{T}\Lambda Q\right)\Sigma {\left(p\right)}^{-1/2}\left(X^{\left(n,p\right)}-np\right)\\ \;={\left(Q\left(\Sigma {\left(p\right)}^{-1/2}\right)\left(X^{\left(n,p\right)}-np\right)\right)}^{T}\Lambda \\ \;\left(Q\left(\Sigma {\left(p\right)}^{-1/2}\right)\left(X^{\left(n,p\right)}-np\right)\right). \end{array}$$

Thus, *Y*^(*n,p*)^ is a quadratic form of the standardized random variable $Q\left(\Sigma {\left(p\right)}^{-1/2}\right)\left(X^{\left(n,p\right)}-np\right)\sim N\left(0,nI\right).$ Letting λ_1_, …, λ_*k*_ be the eigenvalues of Σ(*p*), the distribution of *Y*^(*n,p*)^ is approximated by

(2) $$\begin{array}{l} n\overset{k}{\underset{i=1}{\sum }}\lambda_{i}Z_{i}, \end{array}$$

where the *Z*_*i*_ are independent chi-square random variables with one degree of freedom. It then follows from standard results on quadratic forms (Mathai and Provost, 1992) that

(3) $$\begin{array}{l} 𝔼\left[Y^{\left(n,p\right)}\right]\approx n\text{tr}\left[\Lambda \right]=n\text{tr}\left[\Sigma \left(p\right)\right]=n\overset{k}{\underset{i=1}{\sum }}p_{i}\left(1-p_{i}\right) \end{array}$$

and

(4) $$\begin{array}{l} \text{Var}\left[Y^{\left(n,p\right)}\right]\approx 2n^{2}\text{tr}\left[\Lambda^{2}\right]=2n^{2}\text{tr}\left(\Sigma {\left(p\right)}^{2}\right). \end{array}$$

Although the derivation above required us to assume that the entries of *p* sum to <1, these formula are still useful since for any probability distribution *p* we can obtain an arbitrarily close approximation using the vector *p*′ = (1 − ϵ)*p* for some ϵ > 0. In general, such normal approximations may be less accurate when the entries of *p* are close to zero. However, this will not pose any serious issues for our analysis. For one, this is less problematic in a phylogenetic analysis since the number of sites, *n*, is generally quite large. Secondly, our primary purpose is to gain a better understanding of the meaning of rank-based scores in phylogenetics. As such, we only use the approximation in (2) to obtain estimates for the mean and variance of *Y*^(*n,p*)^ and so that we may apply (Mukerjee and Ong, 2015, Theorem 3), which states that a positive linear combination of independent chi-square random variables has a log-concave cumulative distribution function. This is a result we need in order to prove Lemma 3.3. As the next example shows, these approximations are still quite useful for these purposes.

**Figure 2:**
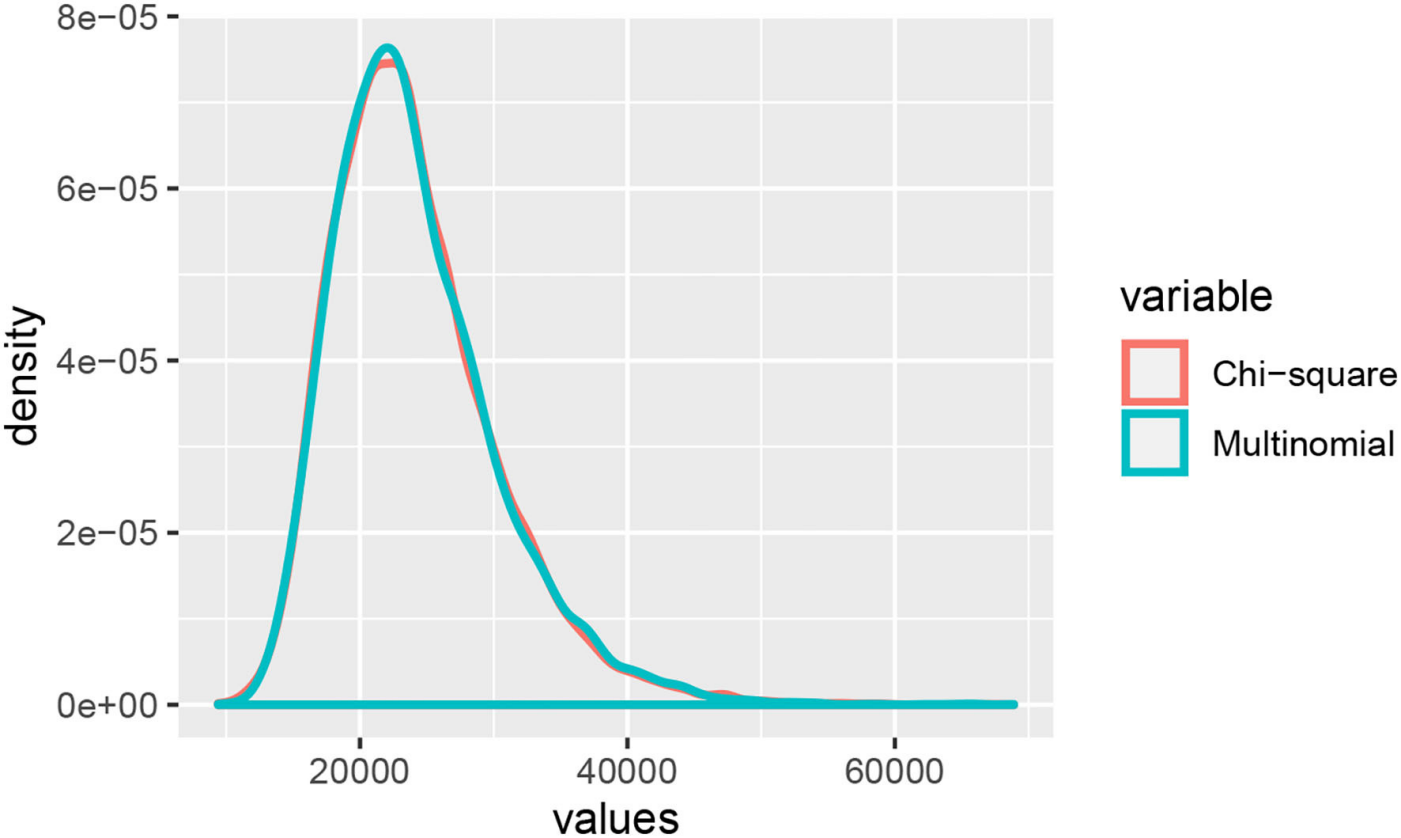
A comparison of samples from *Y*^(*n,p*)^ and the linear combination of chi-squared random variables with a distribution that approximates that of *Y*^(*n,p*)^.

**Figure 3:**
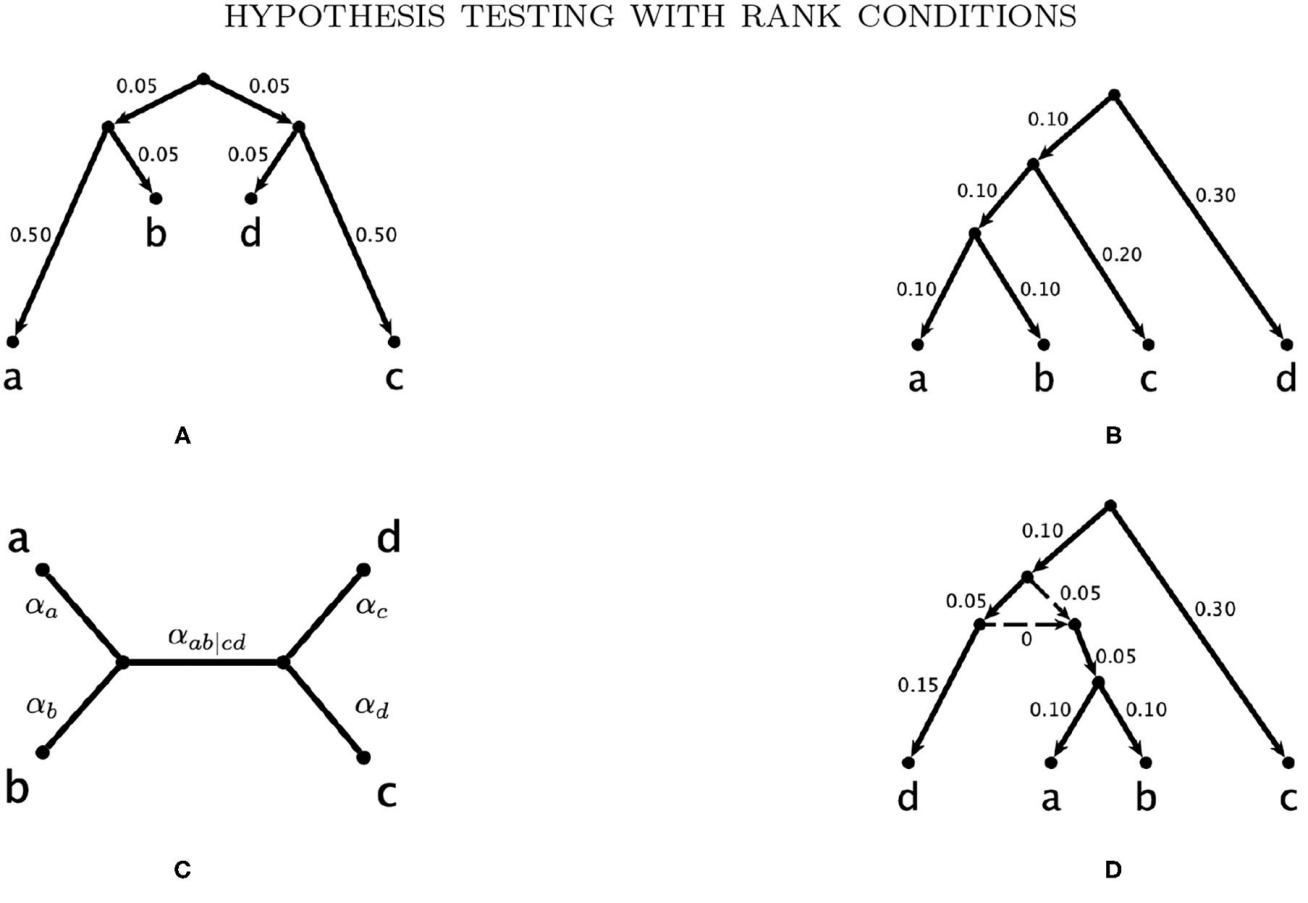
The trees and network used to simulate gene level phylogenies. **(A)** Tree *A*., **(B)** Tree *B*., **(C)** Tree *C*., and **(D)** The network *N* with tree clade *ab*.

**Example 3.1.** Consider the probability distribution *p* from the Jukes-Cantor model on the gene tree ((*a* : 0.5, *b* : 0.05) : 0.05, (*c* : 0.5, *d* : 0.05) : 0.05) (with branch lengths in units of substitutions per site; see the tree in Figure 3A). Figure 2 shows the result of sampling *Y*^(*n,p*)^ via two different methods when the number of sites, *n*, is 25,000.

The “Multinomial” samples are obtained by sampling 25,000 sites from the multinomial distribution *p*, and then computing *Y*^(*n,p*)^. The “Chi-square” samples are obtained by sampling from the distribution approximating *Y*^(*n,p*)^ that was derived in Equation (2) above. That is, we take the linear combination of 256 independent samples from a chi-square distribution where the coefficients are the eigenvalues of Σ(*p*). The densities shown are the result of 10,000 samples. The densities appear quite similar, and the table below shows mean, standard deviation, and .95-quantile for the two samples alongside the theoretical mean and standard deviation computed from the formula above.

**Table:** 

	**Multinomial**	**Chi-square**	**Theoretical**
mean	24068.43	24133.31	24145.84
s.d.	6187.22	6102.42	6183.84
.95-quantile	35395.21	35714.81	–

### 3.2. Bounds on the Mean and Variance of *Y*^(*n,p*)^

In order to obtain bounds for the *p*-values in our hypothesis test, we require bounds on the mean and variance of the test statistic *Y*^(*n,p*)^. In this section, we obtain these bounds using (3) and (4) as approximations of the mean and variance of *Y*^(*n,p*)^.

**Lemma 3.2**. *For all n and all p* ∈ Δ^*k*−1^, *E*[*Y*^(*n,p*)^] ≤ *n and Var*[*Y*^(*n,p*)^] ≤ *n*^2^/2.

*Proof*: First, we have $\text{tr}\left[\Sigma \left(p\right)\right]\leq \overset{k}{\underset{i=1}{\sum }}p_{i}\left(1-p_{i}\right)\leq \overset{k}{\underset{i=1}{\sum }}p_{i}\leq 1$. For the variance, define the function

$$V\left(p\right)=2\text{tr}\left(\Sigma {\left(p\right)}^{2}\right)=2\left(\overset{k}{\underset{i=1}{\sum }}\left(p_{i}^{2}-2p_{i}^{3}\right)+{\left(\overset{k}{\underset{i=1}{\sum }}p_{i}^{2}\right)}^{2}\right).$$

In order to prove the lemma, we will maximize *V*(*p*) over the probability simplex. Thus, we will begin by maximizing *V*(*p*) subject to the constraint *p*_1_ + … + *p*_*k*_ = 1 using Lagrange multipliers. If *k* = 1, *V*(*p*) is always zero, so we assume *k* ≥ 2. Now, to find local optima, we seek *p* ∈ Δ^*k*−1^ and λ ∈ ℝ simultaneously satisfying

$$\begin{array}{l} 2p_{1}-6p_{1}^{2}+4\left(p_{1}^{2}+\ldots +p_{k}^{2}\right)p_{1}-\lambda =0\\ 2p_{2}-6p_{2}^{2}+4\left(p_{1}^{2}+\ldots +p_{k}^{2}\right)p_{2}-\lambda =0\\ \;\vdots \\ 2p_{k}-6p_{k}^{2}+4\left(p_{1}^{2}+\ldots +p_{k}^{2}\right)p_{k}-\lambda =0. \end{array}$$

If such a *p* ∈ Δ^*k*−1^ exists, then for all 1 ≤ *i, j* ≤ *k*, we have

$$\begin{array}{r} 2\left(p_{i}-p_{j}\right)-6\left(p_{i}^{2}-p_{j}^{2}\right)+4\left(p_{1}^{2}+\ldots +p_{k}^{2}\right)\left(p_{i}-p_{j}\right)=0\;\\ \Rightarrow \left(p_{i}-p_{j}\right)\left(2-6\left(p_{i}+p_{j}\right)+4\left(p_{1}^{2}+\ldots +p_{k}^{2}\right)\right)=0. \end{array}$$

Therefore, for each pair 1 ≤ *i, j* ≤ *k*, either *p*_*i*_ = *p*_*j*_ or $\left(2-6\left(p_{i}+p_{j}\right)+4\left(p_{1}^{2}+\ldots +p_{k}^{2}\right)\right)=0.$ If both of the pairs *p*_*i*_, *p*_*j*_, and *p*_*i*_, *p*_ℓ_ satisfy the second equation, then *p*_*j*_ = *p*_ℓ_, which implies that any local optimum can have at most two distinct entries.

First, suppose *p* has only one distinct entry so that *p*_*i*_ = 1/*k* for all 1 ≤ *i* ≤ *k*. Then $V\left(p\right)=\frac{2\left(k-1\right)}{k^{2}}$, which attains its maximum 1/2 when *k* = 2. Now, suppose *p* has two distinct real entries *q*_1_ and *q*_2_ and fix *m* ∈ {1, …, ⌊*k*/2⌋} such that *mq*_1_ + (*k* − *m*)*q*_2_ = 1. Then

$$\begin{array}{r} 2+4\left(mq_{1}^{2}+\left(k-m\right)q_{2}^{2}\right)-6\left(q_{1}+q_{2}\right)=0\;\\ \Rightarrow 4m\left(1+\frac{m}{k-m}\right)q_{1}^{2}-\left(\frac{2m}{k-m}+6\right)q_{1}+\left(2-\frac{2m}{k-m}\right)=0.\\ \Rightarrow \left(2mk\right)q_{1}^{2}+\left(2m-3k\right)q_{1}+\left(k-m-1\right)=0. \end{array}$$

The last line is a quadratic polynomial in *q*_1_, which only has real solutions when

$$\begin{array}{r} {\left(2m-3k\right)}^{2}-4\left(2mk\right)\left(k-m-1\right)\geq 0\;\\ \Rightarrow -4m\left(2k+1\right)\left(k-m\right)+9k^{2}\geq 0. \end{array}$$

Since (*k* − *m*) ≥ *k*/2, if *m* ≥ 3, we have 4*m*(2*k* + 1)(*k* − *m*) > 12*k*^2^ and the inequality is not satisfied. Therefore, the inequality can only be satisfied if *m* = 1 or *m* = 2. In the first case, we have potential local optima where *q*_1_ = 1 or *q*_1_ = (*k* − 2)/2*k*. The value of *V*(*p*) at these points is either 0 or

$$\frac{k^{4}+4k^{3}-16k-16}{8k^{2}{\left(k-1\right)}^{2}}.$$

The latter expression attains a maximum of ≈ 0.43608 when $k=\left(5+\sqrt{13}\right)/3.$ If *m* = 2, we require that −7*k*^2^ + 24*k* + 16 ≥ 0 in order to have a real solution for *q*_1_. This implies that *k* ≤ 4, and since we assumed *m* ≤ ⌊*k*/2⌋, we may assume *k* = 4. In this case, there is a potential local optimum where *q*_1_ = *q*_2_ = 1/4, and *V*(*p*) = 3/8.

We have shown that on the interior of the simplex, every local maximum value of *V*(*p*) is <1/2. In fact, this value is achieved whenever two coordinates are equal to 1/2 and the other *k* − 2 are equal to zero. To find the global maximum of *V*(*p*) over the simplex we must also check the boundary. However, the process of finding the maximum of *V*(*p*) over any face of the simplex amounts to conducting the above analysis for a smaller value of *k*. Therefore, for any *p* ∈ Δ^*k*−1^, Var[*Y*^(*n,p*)^] ≤ *n*^2^/2.     □

### 3.3. Bounds on *p*-Values From the SVD Score

Using the bounds obtained above, we can now state four bounds that can be used to give an upper bound on the *p*-value of our hypothesis tests.

#### 3.3.1. A Bound Using Markov's Inequality

Since *Y*^(*n,p*)^ is a non-negative random variable, we can apply Markov's inequality which gives us that

$$Pr\left(Y^{\left(n,p\right)}\geq \lambda \right)\leq \frac{𝔼\left[Y^{\left(n,p\right)}\right]}{\lambda },$$

for any positive constant λ (Ghosh, 2002).

Combined with Lemma 3.2, this implies that for all *p*,

(5) $$\begin{array}{l} Pr\left(Y^{\left(n,p\right)}\geq \beta \right)\leq \frac{n}{\beta }. \end{array}$$

#### 3.3.2. A Bound Using the Chebyshev Inequality

The one-sided Chebyshev inequality or Cantelli's inequality (Ghosh, 2002) guarantees that for any random variable *X* with mean μ and standard deviation σ and for any λ ∈ ℝ_>0_,

$$Pr\left(X-\mu \geq \lambda \right)\leq \frac{\sigma^{2}}{\lambda^{2}+\sigma^{2}}.$$

By substituting the quantity λ = β − μ, we obtain

$$Pr\left(X\geq \beta \right)\leq \frac{\sigma^{2}}{{\left(\beta -\mu \right)}^{2}+\sigma^{2}},$$

which is valid for any random variable *X* and β > μ. By Lemma 3.2, this implies that for any β > *n* and any *p* ∈ Δ^*k*−1^, we have,

(6) $$\begin{array}{l} Pr\left(Y^{\left(n,p\right)}\geq \beta \right)\leq \frac{\left(n^{2}/2\right)}{{\left(\beta -n\right)}^{2}+\left(n^{2}/2\right)}=\frac{1}{2{\left(\beta /n-1\right)}^{2}+1}. \end{array}$$

#### 3.3.3. A Bound Using a Chebyshev-Like Inequality for Random Variables With Log-Concave CDF

There are various Chebyshev-like inequalities for families of random variables satisfying certain properties such as unimodality or symmetry of the probability density function. In our particular case, we will use an inequality that applies to random variables with log-concave cumulative distribution function. For the following, we will use the approximating distribution (2) for *Y*^(*n,p*)^.

**Lemma 3.3**. *The probability that the random variable Y*^(*n,p*)^ *exceeds β > n is less than or equal to ρ, where ρ is the solution to*

(7) $$\begin{array}{l} \frac{1}{\sqrt{2}\left(\beta /n-1\right)}=\frac{\sqrt{1+2\rho \ln\left(\rho \right)-\rho^{2}}}{\rho -\ln\left(\rho \right)-1}. \end{array}$$

*Proof*: Since *Y*^(*n,p*)^ can be expressed as a positive linear combination of independent chi-square random variables, it has a log-concave cumulative distribution function (Mukerjee and Ong, 2015, Theorem 3). The main theorem in Faridafshin et al. (2017) states that for any random variable with mean μ, standard deviation σ, and log-concave cumulative distribution function, the probability that it exceeds β is less than or equal to ρ, where ρ is the solution to

$$\frac{\sigma }{\beta -\mu }=\frac{\sqrt{1+2\rho \ln\left(\rho \right)-\rho^{2}}}{\rho -\ln\left(\rho \right)-1}.$$

By the bounds in Lemma 3.2, the left hand side of this equation is ≤$1/\left(\sqrt{2}\left(\beta /n-1\right)\right)$ for any *p* ∈ Δ^*k*−1^. Since the right hand side is an increasing function of ρ on [0, 1), the result follows.     □

#### 3.3.4. A Bound Using the Bretagnolle-Huber-Carol Inequality

The ℓ_1_ norm is the vector or matrix norm defined by

$$\|A{\|}_{1}=\underset{i,j}{\sum }|a_{ij}|.$$

We will now apply the following theorem which can be found in van der Vaart and Wellner (1996) to bound $\|X^{\left(n,p\right)}-np{\|}_{1}$.

**Lemma 3.4 (Bretagnolle-Huber-Carol inequality)**. *Let X* = (*X*_1_, …, *X*_*k*_) *be a multinomial random vector with probabilities p* = (*p*_1_, …, *p*_*k*_). *Then for any* λ > 0 *and any sample size n, we have the bound*,

$$Pr\left(\overset{k}{\underset{j=1}{\sum }}|X_{j}-np_{j}|\geq 2\lambda \sqrt{n}\right)\leq 2^{k}\exp\left(-2\lambda^{2}\right).$$

The bound from the inequality above can also be written as

$$Pr\left(\|X^{\left(n,p\right)}-np{\|}_{1}\geq 2\lambda \sqrt{n}\right)\leq 2^{k}\exp\left(-2\lambda^{2}\right),$$

or alternatively, as

$$Pr\left(\|X^{\left(n,p\right)}-np{\|}_{1}^{2}\geq 4\lambda^{2}n\right)\leq 2^{k}\exp\left(-2\lambda^{2}\right).$$

For any matrix or vector *A*, ||*A*||_2_ ≤ ||*A*||_1_, so $Y^{\left(n,p\right)}=\|X^{\left(n,p\right)}-np{\|}_{2}^{2}\leq \|X^{\left(n,p\right)}-np{\|}_{1}^{2}$ and so we have

$$Pr\left(Y^{\left(n,p\right)}\geq 4\lambda^{2}n\right)\leq Pr\left(\|X^{\left(n,p\right)}-np{\|}_{1}\geq 2\lambda \sqrt{n}\right)$$

$$\leq 2^{k}\exp\left(-2\lambda^{2}\right).$$

Substituting $\lambda =\sqrt{\beta /4n}$, this can be rewritten as,

(8) $$\begin{array}{l} Pr\left(Y^{\left(n,p\right)}\geq \beta \right)\leq 2^{k}\exp\left(\frac{-\beta }{2n}\right). \end{array}$$

### 3.4. Comparison of Derived Bounds

In section 3.3, we derived four upper bounds on $Pr\left(Y^{\left(n,p\right)}\geq {\left(S_{r}^{T}\left(\hat{q}\right)\right)}^{2}\right)$, all of which were independent of *p*. Thus, as described, these bounds can be used to obtain an upper bound on

$$\underset{p\in \Delta^{k-1}}{\max}Pr\left(Y^{\left(n,p\right)}\geq {\left(S_{r}^{T}\left(\hat{q}\right)\right)}^{2}\right).$$

For a specific significance level, each of these bounds implies a minimum squared SVD score that will allow us to reject the null hypothesis. That is, for significance level α, we can reject the null hypothesis that $\hat{q}$ is a sample of *n* draws from some $p\in M\subseteq F_{r}^{T}$ if

$$\begin{array}{l} {\left(S_{r}^{T}\left(\hat{q}\right)\right)}^{2}\geq \left(\frac{1}{\alpha }\right)\;n\;(\text{from }(5)),\\ {\left(S_{r}^{T}\left(\hat{q}\right)\right)}^{2}\geq \left(1+\sqrt{\frac{1}{2}\left(\frac{1}{\alpha }-1\right)}\right)\;n\;(\text{from }(6)),\\ {\left(S_{r}^{T}\left(\hat{q}\right)\right)}^{2}\geq \left(\frac{\alpha -\ln\left(\alpha \right)-1}{\sqrt{2\left(1+2\alpha \ln\left(\alpha \right)-\alpha^{2}\right)}}+1\right)\;n\;(\text{from }(7)),\\ {\left(S_{r}^{T}\left(\hat{q}\right)\right)}^{2}\geq -2\ln\;\left(\frac{\alpha }{2^{k}}\right)\;n\;(\text{from }(8)). \end{array}$$

The table below shows the rejection threshold for significance level α that we get from each of the four derived bounds. The rejection threshold is the minimum squared SVD score (divided by *n*) that is required to reject the null hypothesis. Since we are interested in methods of quartet inference on 4-state DNA substitution models, for the Bretagnolle-Huber-Carol (BHC) inequality, we assume that the number of categories is *k* = 256. Notice that for each of the significance levels shown, the bound utilizing the log-concavity of the cumulative distribution function of *Y*^(*n,p*)^ is best.

**Table:** 

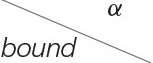	0.10	0.05	0.01	0.001
Markov	10	20	100	1000
Chebyshev	3.24	4.16	8.07	23.36
Log-concave CDF	2.36	2.73	3.68	5.21
BHC	359.50	360.88	364.10	368.70

## 4. Simulations and Results

### 4.1. Power of the Hypothesis Tests

We examine the performance of our hypothesis tests by assessing their power (i.e., the probability that they are able to reject *H*_0_ when it is false) under a variety of scenarios using simulation. Our simulation studies based on gene trees are designed to test the null hypothesis

$H_{0}:\hat{q}$ is a sample distribution obtained by drawing *n* sites from some $p\in M\subseteq F_{4}^{T_{2}}$.

In other words, the null hypothesis is that $\hat{q}$ is a sample from some phylogenetic model on the quartet tree *ac*|*bd* that is contained in the set of rank 4 *ac*|*bd* flattening matrices. The alternative hypothesis for each of our tests is simply that the data were *not* generated by sampling from some $p\in M\subseteq F_{4}^{T_{2}}$. Thus, one way to assess the power of our tests would be to generate data by sampling from some $p\notin F_{4}^{T_{2}}$. This could include, for example, a distribution from one of several phylogenetic models on a discordant gene tree (*ab*|*cd* or *ad*|*bc*) for which it is has been proven that a generic distribution is not contained in $F_{4}^{T_{2}}$ (Allman and Rhodes, 2006). Or, this could include a probability distribution from the multispecies coalescent model on the tree *ac*|*bd*. In this case, the flattening matrix would be expected to be rank 10 rather than rank 4 (Chifman and Kubatko, 2015). Or, we could even sample from any randomly chosen $p\notin F_{4}^{T_{2}}$. It isn't even strictly necessary to sample from a multinomial distribution *p*. Indeed, we could also generate data under a model in which the site patterns were not assumed to be independent and identically distributed. However, we choose to generate data from phylogenetic models where the gene tree or network does not display the split *ac*|*bd*, as these seem like the most likely alternative hypotheses that might be encountered in practice.

In order to assess the power of our tests, we first generate data along the gene trees in Figures 3A,B. We consider two models for nucleotide substitution along the trees, the Jukes and Cantor (1969; JC69) model and the GTR model (Liò and Goldman, 1998), since these span the range of commonly-used empirical models with JC69 being the simplest and GTR being the most complex time-reversible model. In both cases, seq-gen (Rambaut and Grassly, 1997) is used to simulate data along the fixed gene trees in Figures 3A,B. For the JC69 model, the command seq-gen -mHKY is used. For the GTR model, we randomly select rate parameters and base frequencies for each replicate. The rate parameters, *r*_1_, *r*_2_, …, *r*_5_ are sampled from the continuous uniform distribution on the interval (0.5, 1.5) and the base frequency parameters are sampled from a Dirichlet distribution with parameter (5, 5, 5, 5), leading to a mean of 0.25 for each of the base frequencies π_*A*_, π_*C*_, π_*G*_, π_*T*_. The seq-gen command used is thus seq-gen -mGTR -r 1.0 *r*_1_ *r*_2_ *r*_3_ *r*_4_ *r*_5_ -f π_*A*_ π_*G*_ π_*C*_ π_*T*_. The number of sites is varied from 20,000 to 500,000 in increments of 5,000. For each combination of phylogeny, substitution model, and sample size, we repeat the simulation 100 times and record the number of sites required to reached 95% power using each of the tests in section 3.4.

We assess the effect of variation in the branch lengths in two ways. First, we repeat the simulation above using the tree in Figure 3C where all 5 branch lengths (i.e., α_*a*_, α_*b*_, α_*c*_, α_*d*_, and α_*ab*|*cd*_) are randomly sampled from a uniform distribution on the interval (0, 0.1). Simulations are carried out using both the JC69 model and the GTR model with randomly generated parameters, and the number of sites at which the power reaches 95% is recorded in each case. Second, we fix the lengths of the terminal branches in the tree in Figure 3C at either 0.05 or 0.1 and vary the length of the internal branch (α_*ab*|*cd*_) when the sample size is fixed at either 100,000bp or 500,000bp. For each setting, we record the power of each of the tests developed in section 3.4 and display the results in Figure 6.

We then carry out a similar study for the network in Figure 3D, again using the JC69 model and the GTR model with the randomly-selected parameters specified above. We simulate data from the network per the model described in Gross and Long (2018). For this model, a certain portion of the genes are assumed to come from each of the two gene trees created by deleting one of the dotted reticulation edges in the network. For our simulations, half of the data are simulated from each of the constituent trees. The topology of this network comes from Casanellas and Fernández-Sánchez (2020), in which it is also shown that if *p* is a probability distribution arising from this network model, then *F*_*T*_1__ (*p*) will be rank four or less. The branch lengths on the edges of the network were chosen so that each of the constituent trees satisfies the molecular clock.

**Figure 4:**
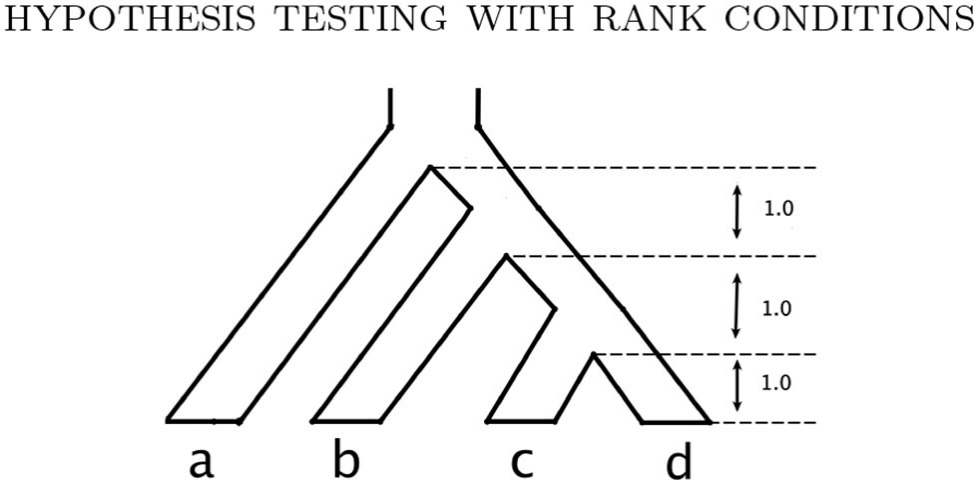
The model species tree used for the simulations under the multispecies coalescent.

Finally, we consider simulating data from the multispecies coalescent model using the species tree shown in Figure 4. For the multispecies coalescent model, the flattening matrix of the true tree is expected to be rank 10 or less (Chifman and Kubatko, 2015), and so we will use the rank 10 SVD scores to test the null hypothesis

$H_{0}:\hat{q}$ is a sample distribution obtained by drawing *n* sites from some $p\in M\subseteq F_{10}^{T_{2}}$.

The ms software (Hudson, 2002) is used to simulate gene trees from the species tree, with the effective population size parameter θ assumed to be constant throughout the tree and set to 0.05 (using the -s 0.1 option in seq-gen). To simulate DNA sequence data along the gene trees generated by ms, we use both the JC69 model and the GTR model with randomly-selected parameters as described above. We consider multilocus data with 100bp per locus and number of loci ranging from 20 to 5,000 in increments of 5. We record the number of sites required to achieve 95% power. Technically, when multiple sites are sampled from the same locus, the multispecies coalescent data cannot be viewed as independent samples from a multinomial distribution, which is one of the assumptions of our hypothesis tests. Still, our simulations indicate that the tests perform as expected, though the required sample sizes are relatively large.

**Figure 5:**
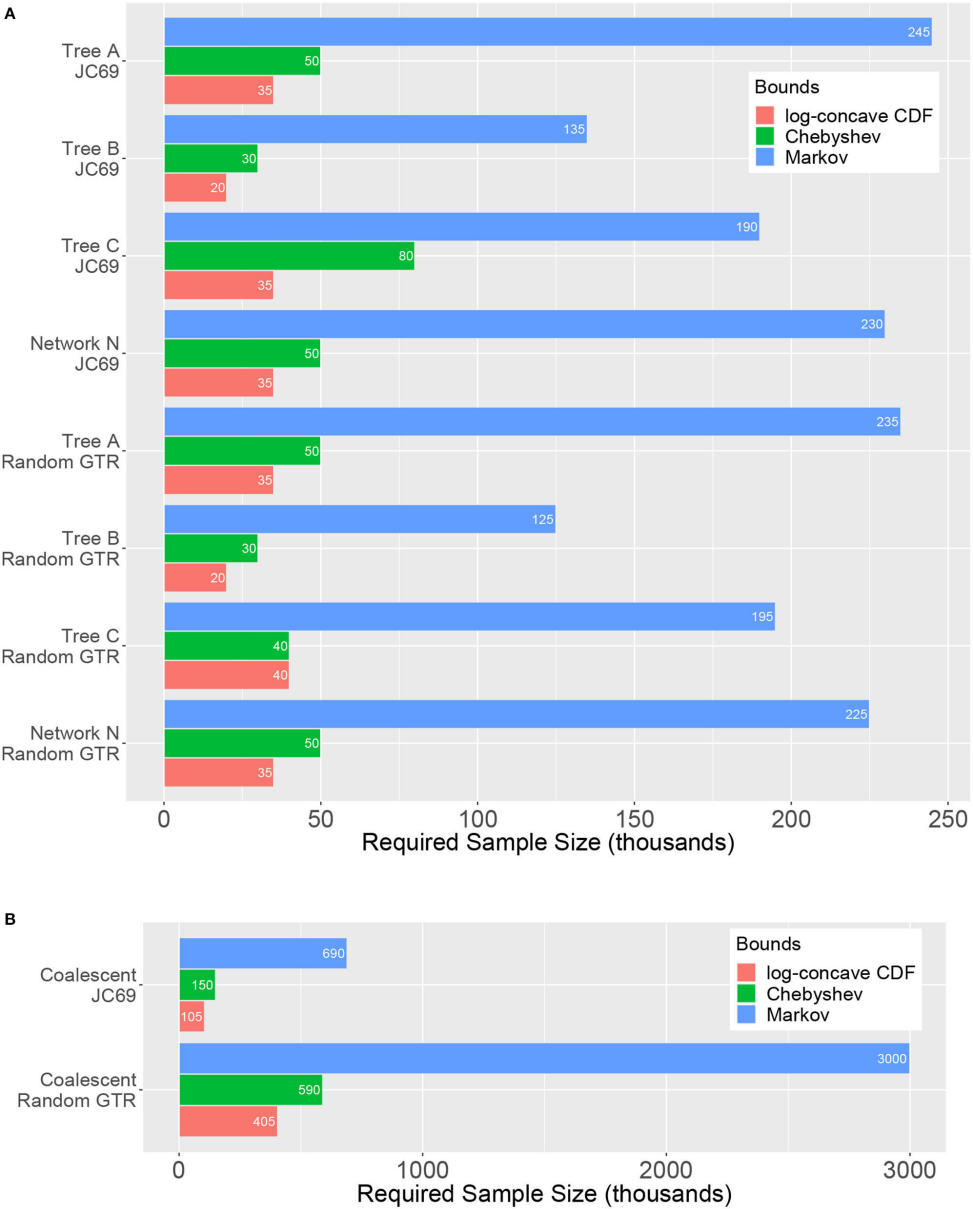
Sample sizes required to achieve 95% power (*x*-axis, in thousands) for various trees and models (*y*-axis) for the hypothesis tests based on the Markov, Chebyshev, and log-concave CDF bounds. **(A)** Gene tree simulations using ${\left(S_{4}^{T_{2}}\left(\hat{q}\right)\right)}^{2}$; **(B)** Coalescent simulations using ${\left(S_{10}^{T_{2}}\left(\hat{q}\right)\right)}^{2}$. Lengths of the bars correspond to the required sample size, and thus shorter bars indicate a more powerful test. The test based on the log-concave CDF bound is clearly the most powerful across the range of conditions explored. The test based on the BHC bound is omitted because it often required sample sizes in excess of 2 million sites to achieve 95% power.

Figure 5 shows the sample size at which the tests first attain 95% power, i.e., the sample size at which at least 95 of the repetitions of the experiment result in an SVD score that is sufficient to reject *H*_0_ at level α = 0.05, for all of the scenarios considered. Results are shown for the test based on the Markov bound, the Chebyshev bound, and the log-concave CDF bound; the BHC bound is not included in the figure as the test based on this bound typically required more than 500,000 sites in order to achieve 95% power (in fact, this test often required more than 2 million sites in order to reliably reject *H*_0_).

It is clear that the test based on the log-concave CDF bound is the most powerful, as predicted by theoretical comparison of the bounds used to derive the test, since it requires the smallest number of sites in order to reject *H*_0_ across all simulation conditions examined. The test based on the Chebyshev bound also performs reasonably well, with required sample sizes just a bit larger then those required for the log-concave CDF bound. By contrast, the test based on the Markov bound performs the worst, often requiring very large sample sizes in order to reliably reject *H*_0_.

Comparing the effect of the nucleotide substitution model, we see that the required sample sizes are generally slightly lower for the random GTR model than for the JC69 model. This might be what one would expect since the JC69 model is a much simpler model of evolution. In fact, for the four-taxon model, there are only 15 unique entries in a length 256 probability distribution vector from the JC69 model. Moreover, it has been shown that after an appropriate change of basis, the concordant flattening matrix will have a block diagonal structure where the blocks satisfy certain rank constraints (Casanellas and Fernández-Sánchez, 2011). Thus, it seems plausible that the many algebraic relationships among the site pattern probabilities for the JC69 model would result in smaller SVD scores for even the discordant flattenings when compared to those for the GTR model with an equal mutation rate. It is also noteworthy that the network requires a similar sample size as the trees to achieve 95% power. We revisit this point later in this section when discussing how we can attain of rough estimate of the number of sites that will be required to reject a certain tree or network.

We would also like to assess the power of our tests by simulating from trees and networks that have an *ad*|*bc* split. For example, we could consider swapping *a* and *c* in the trees and network in Figure 3. However, for Tree *B* and the network *N*, the results will be the same. The reason is that if *p* is a distribution belonging to one of these models, then σ_*ac*_ (*p*), defined by σ_*ac*_ (*p*)_*i*_1_*i*_2_*i*_3_*i*_4__ = *p*_*i*_3_*i*_2_*i*_1_*i*_4__, belongs to the model on the tree or network obtained by swapping the leaves labeled by *a* and *c*. Moreover, *F*_*T*_2__ (σ_*ac*_ (*p*)) can be obtained from *F*_*T*_2__ (*p*) by permuting rows. Since permuting the rows of a matrix does not change the singular values, we will still have $S_{r}^{T_{2}}\left(p\right)=S_{r}^{T_{2}}\left(\sigma_{ac}\left(p\right)\right)$. A similar argument applies when we swap *b* and *d*. In addition, any *p* belonging to the model from Tree *B* or *N* will satisfy *p*_*i*_1_*i*_2_*i*_3_*i*_4__ = *p*_*i*_2_*i*_1_*i*_3_*i*_4__. Consequently, the results will be the same after any permutation of the leaves that yields a tree or network with an *ab*|*cd* or *ad*|*bc* split. This fact also implies that when sampling from Tree *B* or *N*, the expected values of the discordant SVD scores ($S_{r}^{T_{2}}\left(p\right)$ and $S_{r}^{T_{3}}\left(p\right))$) are equal.

Since Tree *A* does not exhibit symmetry between taxa *a* and *b*, the same arguments do not apply, and, we are able to find probability distributions in the models on Tree *A* for which $S_{4}^{T_{2}}\left(p\right)\neq S_{4}^{T_{2}}\left(\sigma_{ab}\left(p\right)\right)$ (or equivalently, for which ($S_{4}^{T_{2}}\left(p\right)\neq S_{4}^{T_{3}}(\left(p\right)$). For this reason, we have included Figure 8 in Appendix A which shows the same results using the other discordant SVD score, $S_{4}^{T_{3}}\left(\hat{q}\right)$. This is equivalent to simulating after relabeling the trees and networks and using the discordant SVD score $S_{4}^{T_{2}}\left(\hat{q}\right)$. As expected, the results are identical for Tree *B* and *N*. They are also identical for Tree *C* since the branch lengths for edges *a* and *b* were chosen randomly in the original simulation. The results for Tree *A* are different, but only very slightly so. Appendix A also includes results from repeating our simulation studies for gene trees with a re-scaling of the entire tree from which the data are simulated using the -s option in seq-gen (-s 0.5 - Figures 9, 10 in Appendix A; -s 2.0 - Figures 11, 12 in Appendix A). Again, each pair of figures shows the results for the two different discordant SVD scores from the same simulations. Finally, Figure 13 in Appendix A shows that we also obtain the same results under the multispecies coalescent model when we use the other discordant SVD score (${\left(S_{4}^{T_{3}}\left(\hat{q}\right)\right)}^{2}$).

Results from the multispecies coalescent simulations are largely consistent with the results from our other simulation studies. We now require more sites to reject the discordant quartets, since the rank 10 SVD scores are smaller than the rank 4 SVD scores we used for gene trees. As in the first case, the test based on the Markov bound often requires very large sample sizes in order to reach 95% power. In contrast to the results for gene trees, however, all three tests are less powerful for data simulated under the GTR model, which is consistent with other simulation studies carried out under the multispecies coalescent (Chifman and Kubatko, 2014). By the same arguments above for gene trees, due to the symmetry between taxa *c* and *d* in the species tree, the results will be the same if we relabel the leaves of this species tree in any way so that it displays either an *ab*|*cd* or *ad*|*bc* split.

**Figure 6:**
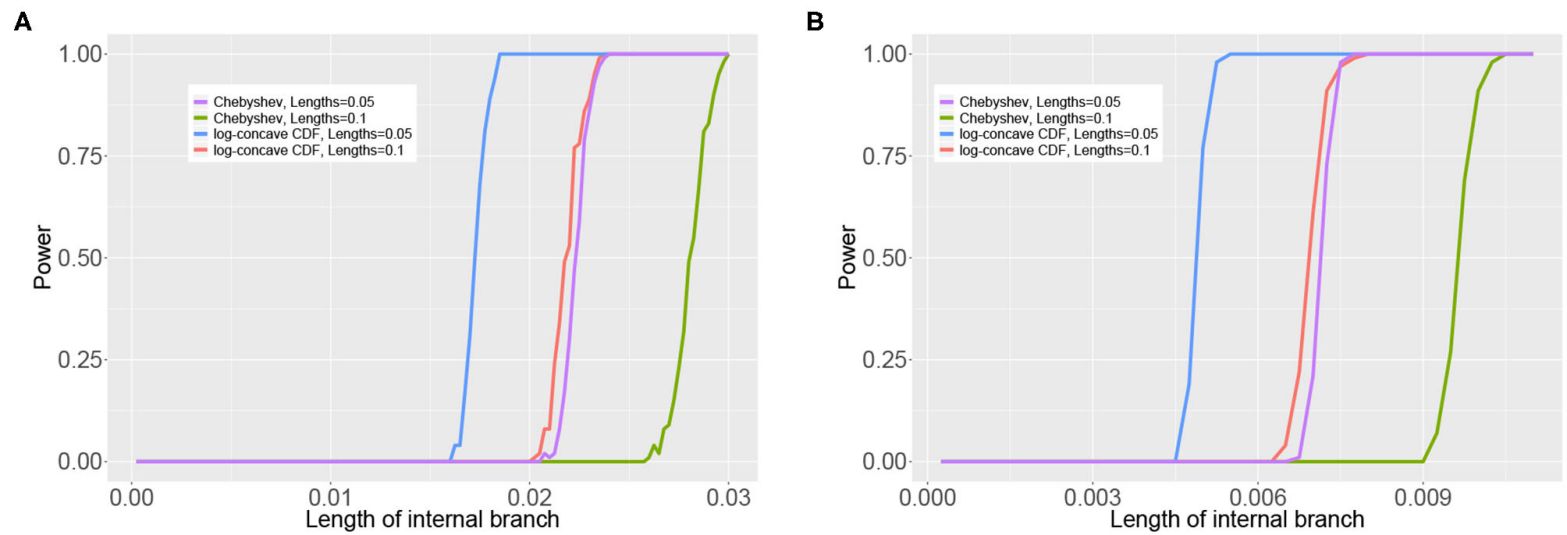
Results of the simulation study that varied the length of the internal branch in the tree in Figure 3C. The *y*-axis shows the power of the tests based on the Chebyshev bound and on the log-concave CDF bound for varying lengths of the internal branch (*x*-axis) using ${\left(S_{4}^{T_{2}}\left(\hat{q}\right)\right)}^{2}$. The sample size was either 100,00 bp **(A)** or 500,000 bp **(B)**. The remaining branch lengths were set to either 0.05 or 0.1.

Figure 6 shows the results of the simulation in which the internal branch in Figure 3C was varied while the terminal branch lengths were held fixed for either 100,000bp or 500,000bp. These simulations again demonstrate that the test based on the log-concave CDF bound is the most powerful, as it allows rejection of the null hypothesis for shorter internal branch lengths for both sample sizes than the test based on the Chebyshev bound. The plots also demonstrate that the power increases with the sample size, as all tests are able to reject the null hypothesis at shorter internal branch lengths when the data consist of 500,000bp.

### 4.2. Statistical Significance vs. Practical Utility

**Figure 7:**
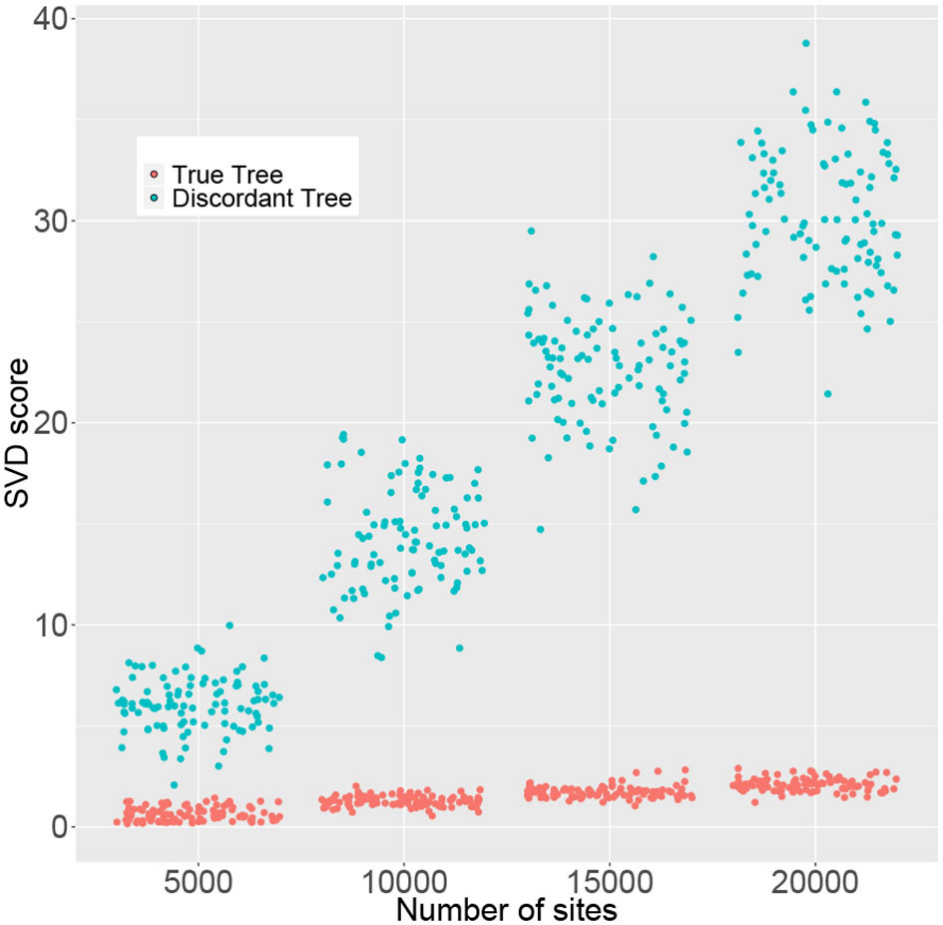
Rank 10 SVD scores (*y*-axis) for 100 replicate data sets computed for the correct tree (*T*_1_, red) and a discordant tree (*T*_2_, green) with varying numbers of sites (*x*-axis). Points were randomly jittered on the x-axis to better display the distribution of SVD scores within each sample size.

The simulation results in the previous section indicate that large sample sizes may be required in some cases in order to reliably reject *H*_0_. However, SVDQuartets is known to perform well for samples that are much smaller than those required to formally reject *H*_0_. To examine this issue in more detail, we simulated data consisting of 50, 100, 150, and 200 genes of length 100 bp under the multispecies coalescent model with the JC69 nucleotide substitution model on the species tree in Figure 4 and we recorded the rank 10 SVD score for both the true tree (*T*_1_) and the discordant tree (*T*_2_). We only show the score for one discordant tree, since as argued above, due to the symmetry in the species tree, for any *p* in the multispecies coalescent model on this tree, the two discordant scores will be equal (i.e., $S_{10}^{T_{2}}\left(p\right)=S_{10}^{T_{3}}\left(p\right)$). The results are shown in Figure 7, where it is clear that the distribution of SVD scores differs substantially between the true tree and the discordant tree, even for the smallest sample size considered (5,000 bp). As the sample size increases, these distributions separate further. Thus, even though statistical significance may not be achieved for small sample sizes, the magnitude of the SVD score may be practically useful for inferring the true phylogeny.

### 4.3. Approximation of Number of Sites Required to Reject

For some of the simpler phylogenetic models, we can obtain precise formulas in terms of the parameters for the theoretical site pattern probability distributions in the model. This allows us to obtain an estimate of the number of sites that would be required to reject a discordant quartet using the SVD score for data generated from a specific distribution in the model. For example, consider the JC69 model for the gene tree ((*a* : 0.5, *b* : 0.05) : 0.05, (*c* : 0.5, *d* : 0.05) : 0.05) shown in Figure 3A. We can compute the theoretical probability distribution for this model exactly and use this distribution to obtain the SVD score for the discordant quartet tree *T*_2_ in Figure 1. The resulting squared SVD score is 8.749732 × 10^−5^. Multiplying a flattening matrix by *c* will multiply the squared SVD score of the matrix by *c*^2^. Thus, if we draw *n* sites from the theoretical distribution, as *n* goes to infinity we expect the squared SVD score to converge to approximately 8.749732 × 10^−5^*n*^2^. Thus, using the log-concave CDF bound with significance level α, we would expect to be able to reject the discordant topology when 8.749732 × 10^−5^*n*^2^ > 2.73*n*, or when *n* is ~31,200 sites. This rough approximation largely agrees with our simulations, which is perhaps not surprising, since the distance to the discordant model is really due to the fact that the model is misspecified.

We can use similar reasoning to estimate the number of sites required to reject an incorrect network topology. Consider first the tree created by removing the reticulation edge of length 0 in the network in Figure 3D. We estimate that it would require 17,000 sites to reject the discordant quartets for data generated under the model on this tree. However, for the tree created by removing the other reticulation edge of the network, we estimate that it would require approximately 58,300 sites to reject the discordant quartets. It seems likely that the longer central edge of the first tree is what makes it easier to reject the discordant quartets, since when the central edge is length zero, all of the quartet flattening matrices will be rank 4 or less. For data from the network, we estimate it would require 28,600 sites to reject the incorrect networks, which is somewhere between the number of sites required for each of the constituent trees.

### 4.4. The SVD Score May Greatly Underestimate the Distance to a Model

By sampling from a known probability distribution in a phylogenetic model, we are able to compare the SVD scores we obtain to the true distance between the expected and observed site pattern frequencies. Consider again the JC69 model on the gene tree ((*a* : 0.5, *b* : 0.05) : 0.05, (*c* : 0.5, *d* : 0.05) : 0.05). When we sample 5000 sites from this distribution, we observe a rather strange phenomena. The rounded distance from the observed to the expected frequency vector is 65.70, while the rounded SVD scores for the three possible quartet trees are shown in the following table.

**Table:** 

Quartet	SVD score
*T*_1_	30.54
*T*_2_	43.65
*T*_3_	50.33

Notice that the distance to the two discordant quartets is actually less than the true distance between the observed and expected site pattern frequency vector. The same phenomena is observed with repeated sampling. This is not necessarily a general phenomenon and depends on the particular choice of parameters and number of sites. If we scale the number of sites by *c*, we expect the true distance from the observed to the expected frequency vector to scale by $\sqrt{c}$ while we expect the discordant SVD scores from the sample to scale by *c*. Consequently, by the same reasoning as in the previous section, we do not expect to regularly observe the same phenomenon if we sample more than around 11,000 sites. Still, this particular example reveals a fundamental limitation in using an SVD score in isolation to reject a particular quartet tree. While the relative order of the SVD scores would lead one to correctly infer that *T*_1_ is the quartet that generated the data, the size of the SVD scores gives a misleading signal about the true distance to the discordant models. We revisit this example in the discussion, but it suggests that the effectiveness of rank-based methods is in the comparison of SVD scores rather than in the SVD scores themselves.

## 5. Discussion

While rank-based methods are extremely effective for phylogenetic inference, our results suggest that the SVD score cannot be readily interpreted as a hypothesis test. The approximations that we make for the test statistic lead to tests that are very conservative in practice and that are prone to Type II errors. Furthermore, if the tests proposed here were to be used in practice, the work of Mitchell et al. (2019) suggests that additional effort may be needed to derive appropriate null distributions for cases that lie near the boundaries between trees (i.e., cases for which the branch lengths are very small). In light of the example discussed in section 4.4, however, it does not appear that there is any significant room for improvement in a similar test based on the SVD score, whether or not a correction for boundary cases is developed. In that example, no similar test could consistently reject one of the discordant quartets, since this would require rejecting the null hypothesis when the discordant SVD score was actually less than the distance between the observed and the expected frequency from the true distribution. Thus, it appears the primary limitation in using the SVD score as a test statistic in phylogenetics is that the SVD score greatly underestimates the distance from an observation to most of the commonly used phylogenetic models.

Similarly, it does not appear that there is much room to improve the bound we obtain in Lemma 3.3. Although one could possibly obtain better bounds by optimizing the formula in Lemma 3.3 over the simplex, the following example shows that this is unlikely to have a significant effect. Consider a hypothesis test of whether an observed site pattern frequency vector came from a particular quartet tree *T*. Let $\tilde{p}$ be the length 256 probability distribution with four entries equal to $\frac{1}{4}$ and all other entries equal to zero. Then

$$Y^{\left(n,\tilde{p}\right)}=\overset{4}{\underset{i=1}{\sum }}{\left(X_{i}^{\left(n,\tilde{p}\right)}-\frac{n}{4}\right)}^{2}.$$

For large *n*, if we divide both sides of this expression by $\frac{n}{4}$, the resulting random variable *Z* follows a χ^2^ distribution with three degrees of freedom. Assuming the chosen significance level is α = 0.05, then we should consider the 0.95 quantile for $Z\sim \chi_{3}^{2}$ which is ≈ 7.815. Thus, the 0.95 quantile of $Y^{\left(n,\tilde{p}\right)}$ is ≈ 7.815*n*/4 = 1.95*n*. Since $\tilde{p}$ has only four non-zero entries, it belongs to $F_{4}^{T}$ for any quartet *T*. Therefore, if ${\left(S_{r}^{T}\left(\hat{q}\right)\right)}^{2}<1.95n$, we cannot reject the quartet *T*. Thus, at least for this significance level, the lowest possible rejection threshold would be 1.95*n*, which is not a substantial improvement over our current lowest threshold of 2.73*n*. Of course, one could reject that the data were generated by $\tilde{p}$ using a different test statistic or the actual distance from the observed data to $\tilde{p}n$. Similarly, one could construct a less conservative test by specifying a particular model and finding the maximum *p*-value over the model. However, our goal here is to take advantage of the speed and elegance of the SVD based methods and to construct a test using only the SVD score.

Though our simulations suggests that the hypothesis tests we develop are not as powerful as might be desired, this work is still useful in further developing our understanding of the SVD score. The fact that the SVD score gives a lower bound on the distance from an empirical site pattern probability distribution to a broad class of phylogenetic models provides some intuition for the disappointing performance of these hypothesis tests. Still, it is possible that the results might be more encouraging on more complex models. As we noted above, for relatively simple phylogenetic models (e.g., the Jukes-Cantor model) there are many algebraic relationships among the site pattern probabilities which may result in lower SVD scores for the discordant flattenings compared to more complex models. This seems to be the case in the gene tree simulations above, where it is actually more difficult to reject the discordant trees under the JC69 model than under the GTR model. Thus, it may be easier to reject the discordant flattenings when the data are generated according to other more complicated models of evolution, e.g., the general Markov Model of sequence evolution (Allman and Rhodes, 2008). Similarly, while examples like the one in section 4.4 demonstrate possible unusual behaviors of the SVD scores, such examples may not necessarily be common across parameter space.

Even though there are limitations to using the SVD score as a hypothesis test, our results clearly demonstrate that the SVD scores encode useful information about which quartet tree generated an observed data set. Comparing SVD scores among trees remains a good method for inferring quartet phylogenies, and thus, of building larger phylogenies under complex models using procedures such as quartet assembly. In addition, the tests derived here may yet prove useful in assigning weights to quartets to provide additional input for quartet assembly algorithms.

## Data Availability Statement

The original contributions presented in the study are included in the article/Supplementary Material, further inquiries can be directed to the corresponding author/s.

## Author Contributions

All authors were responsible for proving the theoretical results, conducting the simulations, and writing the manuscript.

## Conflict of Interest

The authors declare that the research was conducted in the absence of any commercial or financial relationships that could be construed as a potential conflict of interest.
