# Supplementary material for: Hypothesis Testing With Rank Conditions in Phylogenetics
Source: Front Genet. 2021 Jul 2;12:664357. doi: 10.3389/fgene.2021.664357 (PMC8283673; doi:10.3389/fgene.2021.664357)
Supplement: Supplementary file 1 [file Data_Sheet_1.pdf]

Appendix A. Additional Simulation Results

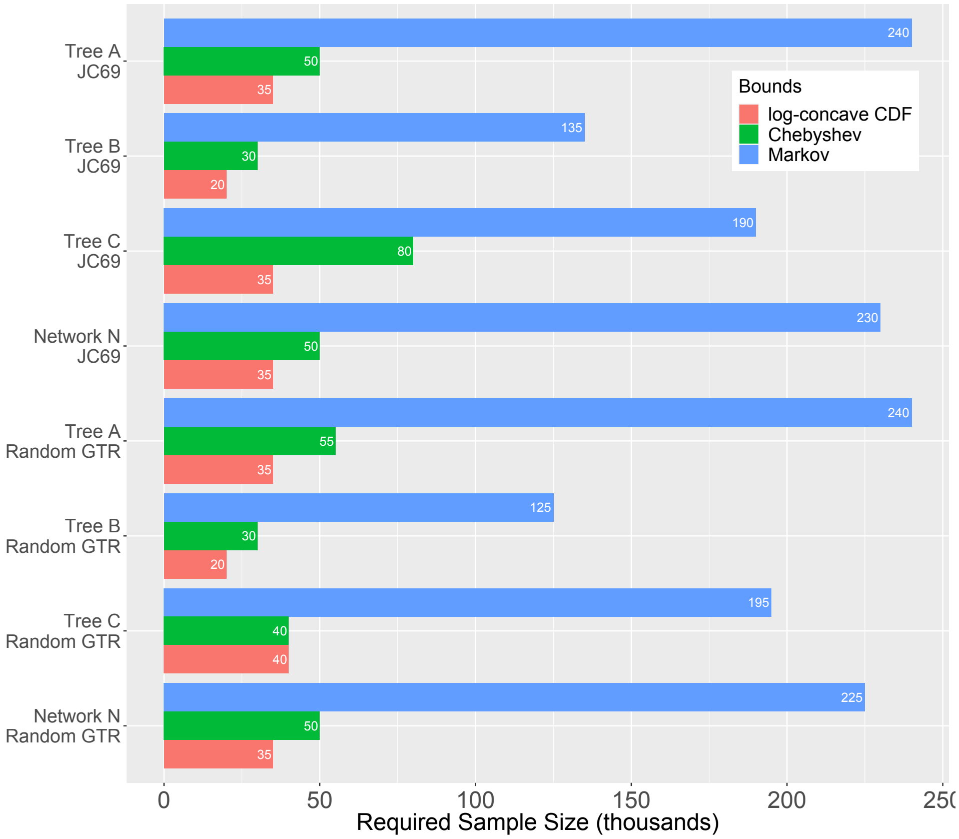

Figure 8. Sample sizes required to achieve 95% power (x-axis, in thousands) for various trees and models (y-axis) for the hypothesis tests based on the Markov, Chebyshev, and log-concave CDF bounds using $(S_4^{T_3}(\hat{q}))^2$. Lengths of the bars correspond to the required sample size, and thus shorter bars indicate a more powerful test. The test based on the log-concave CDF bound is clearly the most powerful across the range of conditions explored. The test based on the BHC bound is omitted because it often required sample sizes in excess of 2 million sites to achieve 95% power.

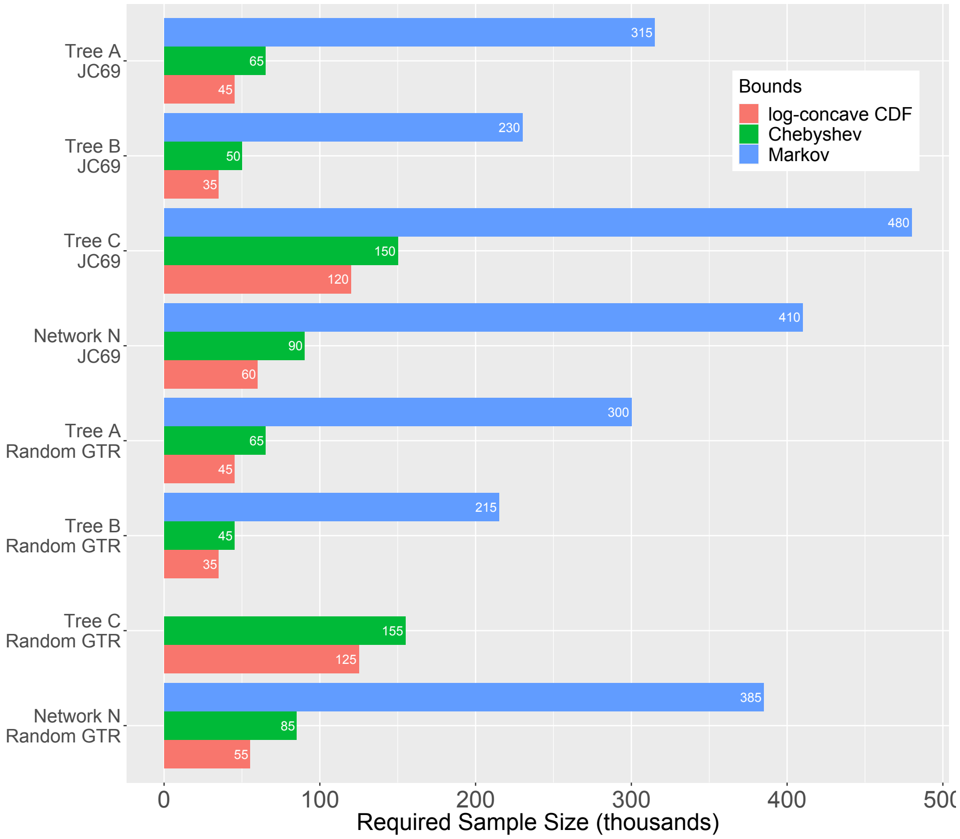

FIGURE 9. Sample sizes required to achieve 95% power (x-axis, in thousands) for various trees and models (y-axis) for the hypothesis tests based on the Markov, Chebyshev, and log-concave CDF bounds using $(S_4^{T_2}(\hat{q}))^2$ when all branch lengths are scaled by a factor of 0.5 for the gene tree simulations. Lengths of the bars correspond to the required sample size, and thus shorter bars indicate a more powerful test. The test based on the log-concave CDF bound is clearly the most powerful across the range of conditions explored. The test based on the BHC bound is omitted because it often required sample sizes in excess of 2 million sites to achieve 95% power. When a bar is not displayed, it indicates that the corresponding test required more than 500,000bp to reach 95% power.

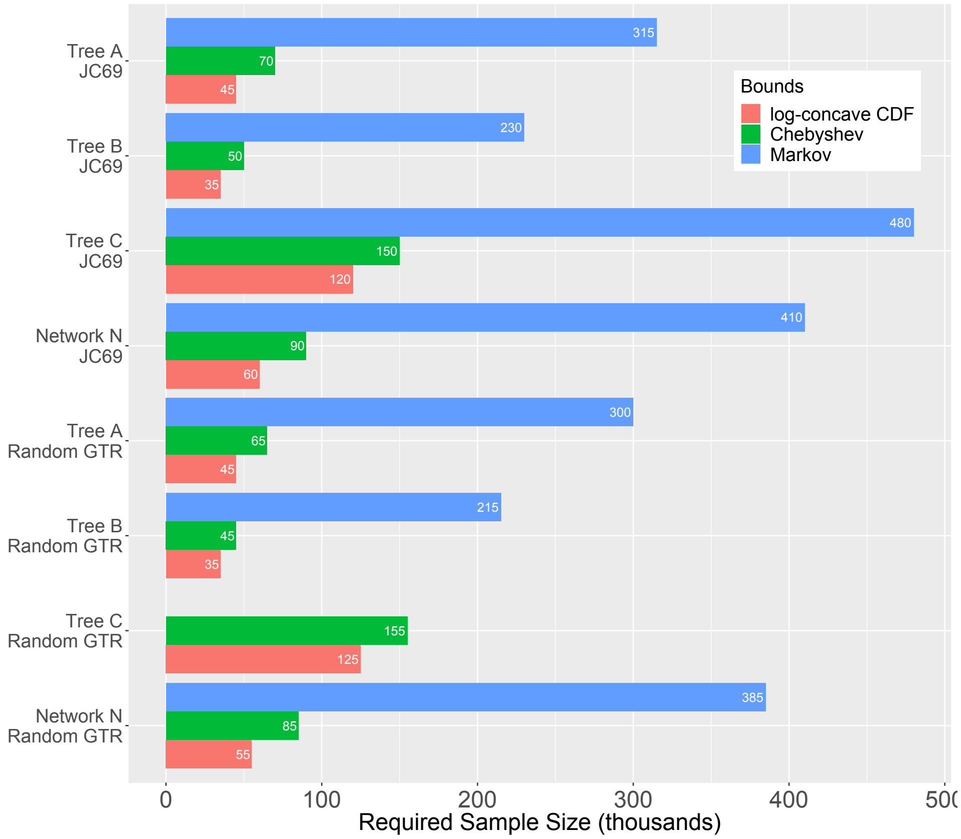

FIGURE 10. Sample sizes required to achieve 95% power (x-axis, in thousands) for various trees and models (y-axis) for the hypothesis tests based on the Markov, Chebyshev, and log-concave CDF bounds using $(S_4^{T_3}(\hat{q}))^2$ when all branch lengths are scaled by a factor of 0.5 for the gene tree simulations. Lengths of the bars correspond to the required sample size, and thus shorter bars indicate a more powerful test. The test based on the log-concave CDF bound is clearly the most powerful across the range of conditions explored. The test based on the BHC bound is omitted because it often required sample sizes in excess of 2 million sites to achieve 95% power. When a bar is not displayed, it indicates that the corresponding test required more than 500,000bp to reach 95% power.

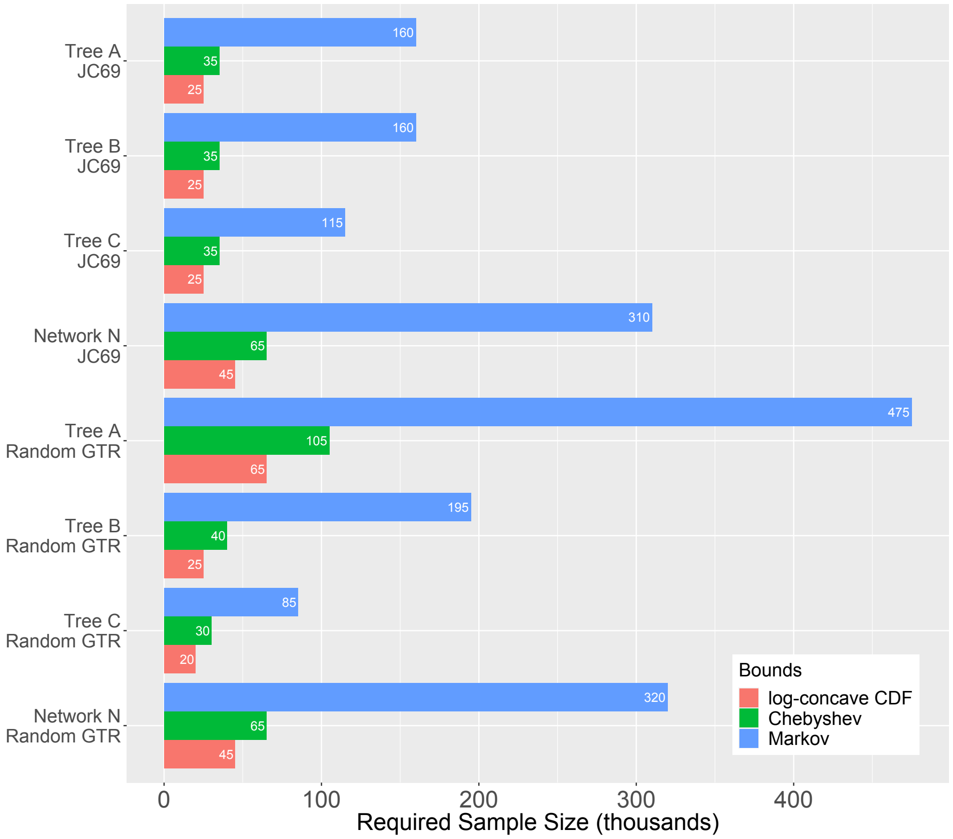

FIGURE 11. Sample sizes required to achieve 95% power (x-axis, in thousands) for various trees and models (y-axis) for the hypothesis tests based on the Markov, Chebyshev, and log-concave CDF bounds using $(S_4^{T_2}(\hat{q}))^2$ when all branch lengths are multiplied by a factor of 2 for the gene tree simulations. Lengths of the bars correspond to the required sample size, and thus shorter bars indicate a more powerful test. The test based on the log-concave CDF bound is clearly the most powerful across the range of conditions explored. The test based on the BHC bound is omitted because it often required sample sizes in excess of 2 million sites to achieve 95% power.

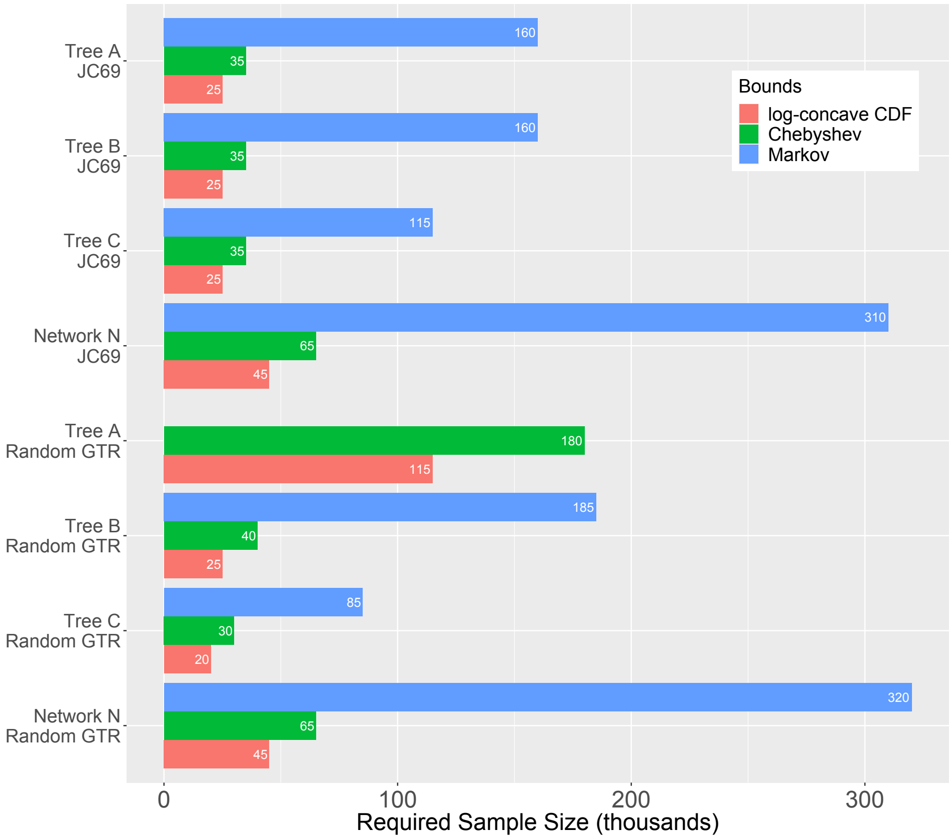

FIGURE 12. Sample sizes required to achieve 95% power (x-axis, in thousands) for various trees and models (y-axis) for the hypothesis tests based on the Markov, Chebyshev, and log-concave CDF bounds using $(S_4^{T_3}(\hat{q}))^2$ when all branch lengths are multiplied by a factor of 2 for the gene tree simulations. Lengths of the bars correspond to the required sample size, and thus shorter bars indicate a more powerful test. The test based on the log-concave CDF bound is clearly the most powerful across the range of conditions explored. The test based on the BHC bound is omitted because it often required sample sizes in excess of 2 million sites to achieve 95% power. When a bar is not displayed, it indicates that the corresponding test required more than 500,000bp to reach 95% power.

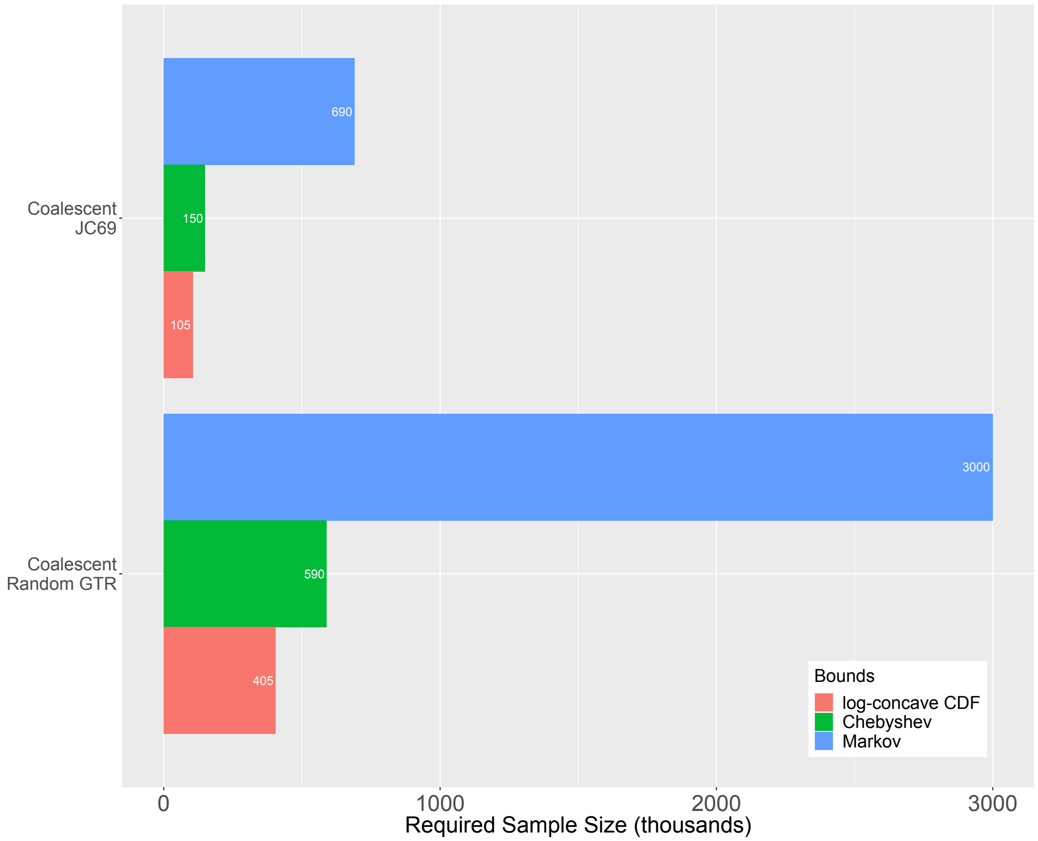

FIGURE 13. Sample sizes required to achieve 95% power (x-axis, in thousands) for various trees and models (y-axis) for the hypothesis tests based on the Markov, Chebyshev, and log-concave CDF bounds using $(S_{10}^{T_3}(\hat{q}))^2$ for the coalescent simulations. Lengths of the bars correspond to the required sample size, and thus shorter bars indicate a more powerful test. The test based on the log-concave CDF bound is clearly the most powerful across the range of conditions explored. The test based on the BHC bound is omitted because it required sample sizes in excess of 5 million sites to achieve 95% power.
